# A comparative study on effects of defect closure versus non-closure in laparoscopic totally extraperitoneal repair of direct inguinal hernia

**DOI:** 10.1097/MS9.0000000000002408

**Published:** 2024-07-23

**Authors:** Vijay Pratap Sah, Bikash Kumar Sah, Nishant Sah, Bhawani Khanal, Abhijeet Kumar, Rakesh Kumar Gupta

**Affiliations:** Department of Surgery, B.P. Koirala Institute of Health Science (BPKIHS), Dharan, Nepal

**Keywords:** defect closure, inguinal hernia, pain, seroma formation, TEP, totally extraperitoneal

## Abstract

**Introduction:**

Totally extraperitoneal (TEP) and transabdominal preperitoneal (TAPP) repair are the principal techniques in laparoscopic hernia repair. Seroma formation and pain are frequent complications of moderate-large size laparoscopic direct inguinal hernia mesh repair. This study was conducted to evaluate the feasibility of defect closure in moderate-large direct inguinal hernias and its effect on various outcomes.

**Method:**

This is a prospective cohort study from September 2020 to August 2021, where a total of 88 patients with uncomplicated direct inguinal hernia (M3 or more) were enrolled in the study and divided into two equal groups of TEP defect closure and non-closure, and various outcome measures were noted.

**Results:**

The majority of patients were male (94.31%), with a mean age range of 18–85 years, and had right-sided inguinal hernia (46.5%). Seroma formation at 10th POD in the defect closure and non-closure were 24% and 33% (*p* value: 0.225), which reduced to 11% and 18%, respectively, at 1 month (*p* value: 1.000). All seromas resolved within 6 months. Pain in VAS at 10th POD in the defect closure and non-closure were 1.55±0.571 and 1.38±0.527, respectively (*p* value: 0.121), which gradually decreased to 1.20±0.524 and 1.16±0.420 at a 6-month interval (*p* value: 0.689). The mean operative time in the bilateral and unilateral defect closure groups was 72.3±4.1 and 56.5±4.3 min, respectively, whereas that in the bilateral and unilateral defect non-closure groups was 62.3±3.7 and 45.7±3.6 min, respectively.

**Conclusion:**

The defect closure was found to have higher pain and less seroma formation at various intervals of time following TEP for moderate-large direct inguinal hernia. Although these findings were statistically insignificant, they may be clinically significant, and further studies with a larger sample size are suggested.

## Introduction

Highlights

- The defect closure in totally extraperitoneal (TEP) repair for moderate-large direct inguinal hernia has less seroma formation.
- The pain was slightly higher in defect closure but statistically insignificant.
- The difference in operative time between the defect closure and non-closure group is statistically insignificant.
- Laparoscopic hernia repair is recommended for bilateral and recurrent inguinal hernias.

Inguinal hernia is a common and widespread condition from which millions of people suffer. Repair of an inguinal hernia is one of the most frequently performed operations in general surgery^1^. European Hernia Society (EHS) has classified groin hernia in the following ways: The size of the hernia orifice is registered as 1 = less than 1.5 cm (1 finger), 2 = less than 3 cm (2 fingers), and 3 = greater than or equal to 3 cm (more than 2 fingers). For the anatomic localization, L = lateral, M = medial, and F = femoral^2^. Totally extraperitoneal (TEP) and transabdominal preperitoneal (TAPP) repair are the principal techniques in laparoscopic hernia repair^3^. Laparoscopic hernia repair is recommended for bilateral and recurrent inguinal hernias. It has also been recommended for patients with primary unilateral inguinal hernia, contingent on the availability of surgical expertise and resources, due to a lower incidence of postoperative pain and chronic pain^4,5^.

Seroma formation is a frequent complication of laparoscopic mesh repair of moderate-large direct inguinal hernia defects. While rates of seroma formation have been reported to be as high as 10–30%^6^. Several attempts have been made to reduce the incidence of seroma formation, such as tacking the transversalis fascia (TF) to the ramus of the pubis, closing the direct inguinal hernia defect via the endoloop technique, and filling the potential dead space with fibrin glue. However, there is a potential increase in the risk of infection and also a risk of chronic pubic bone pain from the tack staples or vasculo-nervous injury if fixing the TF to the abdominal wall, which would lead to extra discomfort for the patient^7^. The closure of a direct hernia defect with a barbed suture not only closes the defect superficially but also exterminates the defect cavity; consequently, the incidence of seroma formation has been greatly reduced^5,8,9^.

However, there is still controversial evidence regarding the choice of the two procedures in terms of reducing the rate of seroma formation and pain. Thus, it is ambiguous which surgical technique should be considered best to repair an inguinal hernia. In this study, we tried to evaluate the technical aspect of direct defect closure in laparoscopic TEP inguinal hernia repair and its effect on the primary outcomes in terms of seroma formation and pain at different time intervals, along with the secondary outcomes such as operative time, length of postoperative hospital stay, days to resume normal activities, recurrence, and intraoperative complications like injury to the vas deference, vessel, and visceral injury or peritoneal tear.

## Methods

It is a prospective cohort study and the work has been reported in line with the STROCSS criteria^10^, which was conducted over a period of 12 months, from September 2020 to August 2021. Ethical approval was obtained from the Institutional Review Committee (IRC /1971/020).

Patients with inguinal hernia presenting to surgery OPD with the inclusion criteria of age greater than 18 years and uncomplicated direct inguinal hernia (≥M3) were enrolled in the study. The exclusion criteria were defect size less than or equal to M2, complicated hernia (irreducible, obstructed, or recurrent hernia), patients unfit for general anesthesia, and patients not giving consent. After obtaining informed written consent a total of 88 patients were enrolled in the study using the purposive alternate number sampling technique. They were divided into 2 groups: 44 patients each in the defect closure and non-closure groups. Postoperative seroma formation and pain are the primary outcomes. All patients were explained and familiarized with the visual analog score (VAS) pain chart preoperatively.

### Sample size calculation

They consider 95% CI and 80% power to estimate the sample size. According to study by Usmani *et al*.[^5^] it was found that the prevalence of seroma formation is 12.6% in defect closure and 6.4% in non-defect closure groups, respectively. Now using the formula-


$$\frac{n=2X(1-\bar{X}\bar{)}{\left(Z\alpha /2+Z\beta \right)}^{2}}{{\left(X1-X2\right)}^{2}=700}$$

Where, *n*=sample size in each group

And, *Zα*/2= 1.96 at 95% CI

*Zβ*= 0.842 at 80% power

*X*1=12.6%

*X*2=6.4%


$$\frac{X\bar{=}X1+X2}{2}$$

Based on last year’s medical records

Total number of laparoscopic inguinal hernia repair at Surgery Department: 100.

Now the study needs corrected sample size formula for finite population (100).

Corrected Sample size formula: Calculated sample size (n_0_) / {1+ Calculated sample size (n_0_) /Sample size of previous year (*N*)}

Corrected sample size formula: [n_0_]/ [1+ (n_0_/*N*)] = 700 / 1+ (700/100) = 88 (44 in each group).

Analyses were performed using SPSS statistical software, version 11.5. Continuous variables were presented as mean and standard deviation and categorical variables as a percentage. Continuous variables were compared between the two groups by Student’s *t*-test and presented as mean ± standard deviation. Categorical variables were compared between two groups by Fischer’s exact test and presented as absolute numbers (percentage).

### Preoperative care

Relevant investigations as per the pre-anesthesia check-up protocol were performed. An informed written consent was obtained. Premedication with benzodiazepine (diazepam 10 mg or lorazepam 2 mg) was done. Patients were kept nil per oral from midnight and were instructed to void just before surgery.

### Intraoperative care

After general anesthesia, the patient was kept in a supine position with both hands tucked. Prophylactic antibiotic (inj. ceftriaxone 1 gram) at the time of induction.

### Operative procedure

TEP repair was performed according to Guidelines for Laparoscopic (TAPP) and Endoscopic (TEP) Treatment of Inguinal Hernia^11^. The operation was performed by the same surgeon throughout the study period using a standard 3-port technique.

All connective tissue was dissected bluntly with a telescope and sharply with a grasper to identify Retzius and Bogros spaces. In direct hernia, content was reduced, and the fascia transversalis (pseudo sac) was pulled and incorporated into closure with a non-absorbable polypropylene barbed monofilament size-0 suture. Then the Parietalisation of the cord structure was done until the peritoneum was reflected to the point at which the vas deference turns medially. Anteriorly, the peritoneum was reflected up to the arcuate line. In patients whose arcuate line was very low, the arcuate ligament was cut 1–2 cm so that an adequate space was created to place the mesh without any wrinkles.

For unilateral inguinal hernia, one polypropylene mesh of standard size 15 ×10 cm was inserted, and for bilateral hernia, two meshes of the same size were inserted, overlapping in the midline in preperitoneal space through the 10-mm port. The two 5-mm port trocars were frequently used for the proper placement and positioning of the mesh. The lower 2–3 cm of the mesh was tucked into the space of Retzius. The mesh was placed without wrinkles to cover all the myopectineal orifices. The space was deflated carefully so as not to displace the mesh.

### Postoperative care

Patient was kept nil per oral and on maintenance intravenous fluid till patient recovers from effects of general anesthesia. The analgesic, ketorolac (30 mg IV), was given at the end of surgery and continued at 8-h intervals postoperatively. Additional requirements for the analgesics (inj. tramadol 50 mg IV with inj. ondansetron 4 mg IV) were given when required. All uncomplicated patients were discharged the next day, as per departmental policy, and encouraged to follow-up in the outpatient department on the 10th day, 1 month, 3 months, and 6 months. At each follow-up pain assessment, seroma formation and hernia recurrence (if any occurred) were noted.

## Results

### Clinic-demographic profile

The mean ages of patients in the defect closure and non-closure groups were 40.13±19.65 and 45.35±18.33 years, respectively. There was no statistical difference in age between the two groups (*p* = 0.153). In the defect closure group, there were 42 males (96.34%) and 2 females (3.7%), whereas in the non-closure group, there were 41 males (94.5%) and 3 females (5.5%) (*P* = 1.000). Most of the patients, 41 (46.5%), had right-sided direct inguinal hernia, 27 (30%) had bilateral direct inguinal hernia, and 20 (25%) had left-sided direct inguinal hernia, as shown in (Table 1).

**Table 1:** Clinic-demographic profile

S.no.	Characteristics	defect closure	Non-defect closure	*P*
1.	Age (years)	18–85	18–81	0.153
2.	Sex, *n* (%)			
	Male	42 (96.4)	41 (94.5)	1.000
	Female	2 (3.6)	3 (5.5)	
3.	Hernia side			
	Right	18	23	
	Left	11	9	
	Bilateral	15	12	

### Intraoperative characteristics

Operative time in the defect closure group was slightly higher than that of the non-closure group in unilateral and bilateral TEP repair, but the difference was not statistically significant. Also, there was no significant difference in the intraoperative complication rate in terms of vas deference, vessel and visceral injury, or peritoneal tear between the two groups, as shown in (Table 2).

**Table 2:** Intraoperative characteristics

S.no.	Intraoperative characteristics	Defect closure	Non-defect closure	*P*
1.	Operative time (in min)			
	Unilateral	56.5±4.3	45.7±3.6	0.621
	Bilateral	72.3±4.1	62.3±3.7	0.513
2.	Vessels injury, *n* (%)	2 (2)	3 (3)	0.500
3.	Peritoneal tear, *n* (%)	7 (16)	4 (11)	0.580
4.	Visceral injury	No	No	
5.	Vas deference injury	No	No	

### Postoperative outcome

In our study, there was no significant difference in hospital stay or resume of normal activity. Pain was slightly more common in the defect closure group but was statistically insignificant. Seroma formation was less in the defect closure group but was statistically insignificant. In the 6 months of follow-up, no one had seroma formation. Also, there was no hernia recurrence within 6 months of the interval, as shown in (Table 3).

**Table 3:** Postoperative outcomes

S.no.	Postoperative outcomes	Defect closure	Non-defect closure	*P*
1.	Postoperative hospital stays (days)	1.16±0.420	1.2±0.447	0.661
2.	Resume normal activity (days)	7.15±1.129	7.18±1.565	0.889
3.	Pain in VAS			
	10 days	1.55 ± 0.571	1.38 ± 0.527	0.121
	1 month	1.25 ± 0.615	1.2 ± 0.447	0.596
	3 months	1.24 ± 0.607	1.11 ± 0.369	0.188
	6 months	1.20 ± 0.524	1.16 ± 0.420	0.689
4.	Seroma formation, *n* (%)			
	10 days	13 (24)	18 (33)	0.225
	1 month	6 (11)	10 (18)	1.000
	3 months	0	1 (1)	0.500
	6 months	0	0	0.000
5.	Recurrence within 6 months	0	0	0.000

VAS, visual analog score.

## Discussion

This study was conducted to evaluate the effect of laparoscopic defect closure in moderate-large (≥M3) direct inguinal hernias. IEHS/EHS Bittner *et al*.^11^. also recommend defect closure in cases of large direct inguinal hernia (M3), as it prevents seroma formation, infection, recurrence, and chronic pain, altogether reducing discomfort and postoperative complications.

### Age distribution

In our study, most of the patients were in the middle age group, with a mean age of 40.13±19 and 45.35±18.33 years in the defect closure and non-closure groups, respectively. Similarly, in studies by Parshad *et al*.^12^. and Garg *et al*.^13^. the mean age was 46.40 and 47.16 years, and 56.8 and 45.27 years in the defect closure and non-closure groups, respectively. The results showed consistency of mean age in studies done at various geographical locations, suggesting that the incidence of direct hernia occurs mostly after the 4th decade of life, as with increasing age, the weakness in the abdominal wall musculature increases.

### Operative time

The reasons behind more operative time in defect closure are due to the time taken during suturing the defect (~ 4–5 bites). The operative time in our study was consistent with other studies (Table 4).

**Table 4:** Operative time

		Operative time		
Study	Year	Defect closure	Non-closure	*P*
Zhu *et al*.^14^	2018	55.0+8.09	44.5+9.5	0.001
Kane *et al*.^15^	2018	91.1+37.1	76.8+32.9	0.001
Usmani *et al*.^5^	2019	91.9+41.3	86.7+35.7	0.523
Our study	2021	56.5+4.3	45.7+3.6	0.513

### Vessel injury

Our study found bleeding from the inferior epigastric artery (IEA) in 5.5% of cases, which is almost double that mentioned by Bittner *et al*.^10^. The higher incidence may be due to the operating surgeon’s initial phase of learning. The bleeding was controlled by either clip, monopolar, or ligature energy devices.

### Peritoneal tear

The incidence of peritoneal tear was higher, that is 47%, in the study by Lau *et al*.^16^. in relation to our study. The techniques for the closure of a peritoneal tear include pretied suture, loop ligation, endoscopic stapling, and endoscopic suturing. Careful dissection in close proximity to the vas deferens, in addition to cautious use of traction and countertraction, associated with prudent application of sharp dissection with endo-scissors to divide adhesions, can help to prevent peritoneal laceration.

### Seroma formation

The incidence of seroma formation was slightly higher in the non-closure group as compared to the defect closure group, although it was statistically non-significant. Similar findings were reported by various studies, as shown in (Table 5), except for a study by Koch *et al*.^17^. In closing the wall of the direct defect, the pulled fascia transversalis was also incorporated. This led to obliteration of the empty cavity of the defect, hence less seroma formation. Thus, a large defect size with huge dead space, a wider dissection area, inguinoscrotal hernia, and a residual hernia sac result in more seroma formation.

**Table 5:** Seroma formation

			Defect closure		Non-closure	
Author	Year	Follow-up in months	Seroma	Total	Seroma	Total
Parshad *et al*.^12^	2005	20	2	29	4	34
Koch *et al*.^17^	2006	30	10	26	2	27
Reddy *et al*.^18^	2007	28	4	96	5	35
Garg *et al*.^13^	2011	25	5	52	8	52
Li j, Zhang^8^	2016	9	1	36	NA	NA
Zhu *et al*.^14^	2018	4	4	30	22	30
Usmani *et al*.^5^	2019	51	14	219	20	159
Our study	2021	6	13	44	18	44

NA, not applicable.

All the seromas were managed conservatively, except in 2 patients at 1 month of their presentation, as patients were experiencing discomfort, so the seroma was aspirated under aseptic precautions.

### Postoperative hospital stay

In our study, there was no significant difference in postoperative hospital stay between both groups. It was similar to the study conducted by Garg *et al*.^13^. which showed 1.12±0.3 days in the defect closure group and 1.12±0.4 days in the non-closure group. There are many studies, such as those by Usmani *et al*.^5^., Li *et al*.^8^., Zhu *et al*.^14^, Parshad *et al*.^12^, Koch *et al*.^17^, Reddy *et al*.^18^. mentioning no differences between the two groups for postoperative hospital stays.

### Days to return to normal activities

As in any laparoscopic surgery, our study reports an early return to work, similar to the study by Garg *et al*.^13^.

### Pain

Although the finding is statistically not significant, our study showed slightly higher pain in the defect closure group at 10th POD, 1 month, 3 months, and 6 months POD in comparison to the non-defect closure group. A similar finding has been reported in a study by Koch *et al*.^17^, where the pain in VAS was 0.8±1.7 and 0.3±0.8 in defect closure and non-closure groups, respectively, with a *p* value of 0.15, which was statistically not significant. The reasons for pain may be due to deep suited bite of the suture or the nerve entrapment by the suture, such as osteitis pubic.

## Limitation

There are a few limitations to this study, the first being the small sample size of 88. The reliability of the measured complications would be improved with a larger sample size. Our follow-up of both groups was limited to 6 months, where assessment of only early recurrence was feasible. Surgery was performed by a single surgeon whose experience may limit the ability to generalize the result of an entire population undergoing TEP repair. There was no preoperative pain assessment.

## Conclusion

The defect closure was found to have higher pain and less seroma formation at various intervals of time following TEP for moderate-large direct inguinal hernia. Although these findings were statistically insignificant, they may be clinically significant. And there was no statistical difference in the secondary outcome of defect closure or non-closure in terms of operative time, length of postoperative hospital stays, days to resume normal activities and work, recurrence, or intraoperative complications like Vas deference, vessel, visceral injury, or peritoneal tear.

## Consent

Written informed consent was obtained from the patient for publication and any accompanying images. A copy of the written consent is available for review by the Editor-in-Chief of this journal on request.

## Source of funding

None.

## Author contribution

V.P.S.: concept, design, definition of intellectual content, literature search, clinical studies, data acquisition, data analysis, statistical analysis, manuscript preparation, manuscript editing, manuscript review. B.K.S.: concept, design, definition of intellectual content, literature search, clinical studies, data acquisition, data analysis, statistical analysis, manuscript preparation, manuscript editing, manuscript review. N.S.: concept, design, definition of intellectual content, literature search, clinical studies, data acquisition, data analysis, statistical analysis, manuscript preparation, manuscript editing, manuscript review. B.K.: concept, design, definition of intellectual content, literature search, clinical studies, data acquisition, data analysis, statistical analysis, manuscript preparation, manuscript editing, manuscript review. A.K.: concept, design, definition of intellectual content, literature search, clinical studies, data acquisition, data analysis, statistical analysis, manuscript preparation, manuscript editing, manuscript review. R.K.G.: concept, design, definition of intellectual content, literature search, clinical studies, data acquisition, data analysis, statistical analysis, manuscript preparation, manuscript editing, manuscript review.

## Conflicts of interest disclosure

The authors declare no conflicts of interest.

## Research registration unique identifying number (UIN)

Registry used- Clinical trials registry.

Registration ID: NCT06389331 https://clinicaltrials.gov/study/NCT06389331?cond=Study%20on%20Effects%20of%20Defect%20Closure%20in%20Laparoscopic%20Repair%20of%20Direct%20Inguinal%20Hernia&rank=1.

## Guarantor

Vijay Pratap Sah and Abhijeet Kumar.

## Data availability statement

Datasets generated during and/or analyzed during the current study are available upon reasonable request.

## Provenance and peer review

Not commissioned, externally peer-reviewed.
